# 1,1,6-Trimethyl-1,2-dihydronaphthalene (TDN) Sensory Thresholds in Riesling Wine

**DOI:** 10.3390/foods9050606

**Published:** 2020-05-09

**Authors:** Andrii Tarasov, Nicoló Giuliani, Alexey Dobrydnev, Christoph Schuessler, Yulian Volovenko, Doris Rauhut, Rainer Jung

**Affiliations:** 1 Department of Enology, Hochschule Geisenheim University (HGU), Von-Lade-Straße 1, 65366 Geisenheim, Germany; nicolo.giuliani@live.com (N.G.); Christoph.Schuessler@hs-gm.de (C.S.); Rainer.Jung@hs-gm.de (R.J.); 2 Department of Organic Chemistry, Faculty of Chemistry, Taras Shevchenko National University of Kyiv, Volodymyrska str. 60, 48000 Kyiv, Ukraine; alexey.pierrot@gmail.com (A.D.); yulian_volovenko@ukr.net (Y.V.); 3 Department of Microbiology and Biochemistry, Hochschule Geisenheim University (HGU), Von-Lade-Straße 1, 65366 Geisenheim, Germany; Doris.Rauhut@hs-gm.de

**Keywords:** 1,1,6-Trimethyl-1,2-dihydronaphthalene (TDN), wine, sensory threshold, serving temperature

## Abstract

1,1,6-Trimethyl-1,2-dihydronaphthalene (TDN) is an aroma compound responsible for the kerosene/petrol notes in Riesling wines. In the current article, three sensory thresholds for TDN were determined in young Riesling wine: *detection threshold* (about 4 µg/L), *recognition threshold* (10–12 µg/L), and *rejection threshold* (71–82 µg/L). It was demonstrated that an elevated content of free SO_2_ in wine may have a certain masking effect on the TDN aroma perception. In addition, the influence of wine serving temperature on the recognition of kerosene/petrol notes was studied. It was found, that a lower wine serving temperature (about 11 °C) facilitated identification of the TDN aroma compared to the same wine samples at room temperature.

## 1. Introduction

1,1,6-Trimethyl-1,2-dihydronaphthalene (TDN) is one of the key wine aroma components in Riesling wines, and it belongs to the C_13_-norisoprenoids. With the kerosene/petrol aroma, TDN is considered controversial from the consumers’ preference perspective. Low and medium TDN concentrations contribute to the complexity of the wine bouquet, while high TDN content often evokes negative impressions caused by the strong kerosene/petrol odor dominance.

The level of TDN in wine increases during bottle aging due to the transformations of carotenoid-derived precursors originating from grapes [1,2,3]. The quantity of TDN precursors depends on the viticulture practices such as grape clusters defoliation [4,5], soil fertilization [6], water irrigation [7,8], and the selection of vine clones [9]. Global climate change, warmer temperatures, and higher sun exposure of the grapes may intensify formation of TDN in the succeeding Riesling wines [10,11]. The option of yeast strains can also affect the formation of TDN in wine, probably due to the pathways of the precursors’ conversion [9,12]. The TDN level in wine can also be managed by the selection of bottle closures, which are able to absorb a significant amount of TDN from the wine [13,14,15,16]. Finally, it was demonstrated that wine storage conditions, e.g., elevated temperature, can accelerate the formation of TDN [17,18].

The typical content of TDN in European Riesling wines is usually between 1 and 50 μg/L, while in Australian wines it can reach up to 250 μg/L and more [19,20,21,22]. The sensory threshold of TDN in wine was defined in several studies as being in a range of values between 2 and 20.6 μg/L (Table 1). In the first publication, Simpson (1978) reported the *flavor threshold* in Riesling at 20 μg/L, however, no details regarding the panel were described. Several decades later, the TDN *odor detection threshold* (*ODT*) was determined at a significantly lower level, 2 μg/L [20]. Trained panelists evaluated model and Chardonnay spiked wines in a series of 3-AFC tests. The succeeding work also utilized 3-AFC tests but with an untrained panel of consumers using spiked Riesling wines. As a result, the defined *ODT* values were close to the initial one, about 20 μg/L [23]. In addition, the *consumer rejection threshold* (*CRejT*) in this research was also determined to be 157 μg/L and 82.4 μg/L depending on the wine vintage (2010 and 2011) and the country in which the tests were conducted (New Zealand and the USA), respectively. A recent study revealed the following values of TDN perception thresholds in Riesling wine: 3.1 μg/L for *ODT* by trained panellists and 14.7 μg/L for *consumer detection threshold* (*CDT*) [24]. In the same work the *CRejT* was found to be 60 µg/L and 91 µg/L for young and aged Riesling wines, respectively.

The variability of the reported TDN sensory thresholds values can be related to both diverse concepts of sensory thresholds and variations in sensory evaluation methods. In the current work, we aimed to define and study *detection* and *recognition thresholds* of TDN:

- *detection threshold* (*DT*) implies the lowest level at which a stimulus can be detected, but not necessarily recognized;
- *recognition threshold* (*RT*) corresponds to the level when a stimulus can be recognized and identified; it is usually higher than *DT* [25].

In addition our goal was to determine a TDN *rejection threshold* (*RejT*) and to compare it with the previously reported *CRejT* values. This issue was of interest since various approaches to the evaluation of *rejection thresholds* are still under discussion [26].

Finally, this study investigated the influence of free SO_2_ levels and wine serving temperature on the perception of TDN aroma. These factors can vary significantly in reality and, to our knowledge, have not been previously studied. Earlier, it was demonstrated that ethanol levels and carbonation can enhance the *odor detection threshold* of TDN in certain matrices [24].

## 2. Materials and Methods 

### 2.1. Chemicals and Materials

The following chemicals were used for the experiment and analyses: ethanol absolute AnalaR NORMAPUR^®^ ACS, ≥99.5% (VWR Chemicals); sodium chloride (Carl Roth GmbH, Germany); *β*-ionone-*d_3_*, ≥95% (aromaLAB GmbH, Germany); and 1,1,6-trimethyl-1,2-dihydronaphthalene ≥ 95% (own synthesis [27]). Parafilm “M”^®^ was purchased from Carl Roth GmbH, Germany.

Transparent green glass bottles (1 L volume) with MCA finish type were supplied by Richard Wagner GmbH + Co. KG, Alzey (Germany). Screw caps of MCA type were supplied by Rheingauer Winzerbedarf GmbH. The base wine, Riesling Villa Monrepos from the Rheingau region (Germany) of 2016 vintage, was bottled in April 2017 in the winery of the Hochschule Geisenheim University. Analysis of the wine after the bottling revealed the following: alcohol content 12.3% (*v*/*v*), titratable acidity 7.3 g/L, sugar content 7.5 g/L, and pH 3.2. The TDN concentration in the wine was 2.2 µg/L (analysis before the *sensory sessions*). The adjustment of the SO_2_ content in the wine resulted in free/total SO_2_ concentrations of 40/120 mg/L for *Sensory Session 1* (high free SO_2_ content) and 10/65 mg/L for *Sensory Session 2* (low free SO_2_ content). The high free SO_2_ content of 40 mg/L is relatively elevated compared to many wines globally, but is typical for many Riesling wine producers in the Rheingau region. This particularity is explained by the local practices and expectations of a longer aging time of these wines.

Young Riesling wine, 2016, was selected as the base wine for all the sensory tests in order to avoid an elevated initial level of TDN. Wines produced from other international grape varieties were not used since they typically do not possess noticeable amounts of TDN [20] and kerosene/petrol aroma. Furthermore, the composition of other wine matrices may affect the TDN perception thresholds values, which makes them inapplicable for Riesling wines.

The free SO_2_ content in wine is one of the parameters that can have an impact on the wine aroma perception. The influence of *low* and *high* free SO_2_ content on TDN recognition in Riesling wine was studied in the current research, since no information on this issue was found in previous publications. The same Riesling wine with *high* (40 mg/L) and *low* (10 mg/L) content of free SO_2_ was used in the tests of *Sensory Sessions 1* and *2*, respectively. 

### 2.2. Preparation of TDN Stock Solutions

The TDN stock solution was prepared by the addition of 9.1 mg of TDN into a 50 mL volumetric flask, which was then filled with ethanol to the 50 mL mark, resulting in a TDN concentration of about 0.182 mg/mL. The stock solution was stored in a refrigerator at 4 °C with a ground glass stopper, additionally sealed with Parafilm^®^. Before the wine spiking procedures, the TDN stock solution was kept for about 15–30 min outside the refrigerator at room temperature.

### 2.3. Panels for Sensory Sessions

Two panels participated in the *sensory sessions*. Panel 1 (*Sensory Session 1*) consisted of 20 tasters: 11 male and 9 female. Panel 2 (*Sensory Session 2*) comprised 22 tasters: 13 male and 9 female. The age of the tasters was in the range between 21 and 45 years. All the panelists, employees or students of the Hochschule Geisenheim University (Germany), were regular wine consumers. Both panels were international (more than 15 nationalities from European, Asian, and the American countries). All the tasters were familiar with Riesling wines and their typical aromas, therefore, no special training or panelists selection was done. The sensory evaluations were conducted in June 2017 in the specialized well-lit (white light) and odor-free sensory analysis room in the Department of Enology of the Hochschule Geisenheim University (Germany). There were 30 separated booths, specialized individual places for panelists. The room temperature was about 22 °C. 

**Figure 1:**
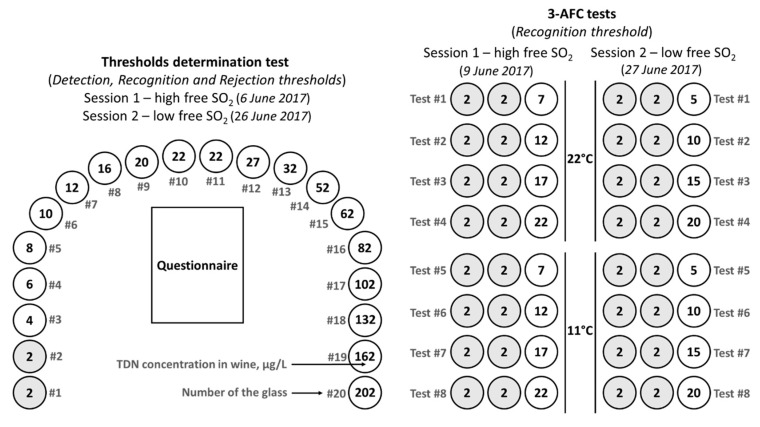
Design of the experiment. Scheme of the glasses with TDN spiked Riesling wines presented for the *sensory sessions*. The gray background of the circle indicates the base wine without TDN spiking.

Each *sensory session* consisted of two parts: *thresholds determination test* (for *DT*, *RT,* and *RejT*) and a *series of 3-AFC tests* (Figure 1). The structure and content of both *sensory sessions* were identical except for the level of free SO_2_ in the wine samples. The evaluation of the wine samples in all the tests was orthonasal. 

### 2.4. Thresholds Determination Tests

The *thresholds determination test* [28] was preferred to the 3-AFC test methods (according to ISO 13301:2018 [29] and ASTM (American Society for Testing and Materials) E679-19 [30]) for two reasons. First, we aimed to compare our sensory thresholds outcomes with the previously reported values defined by the 3-AFC test methods (Table 1). Second, the offered approach allowed a convenient determination of the three sensory thresholds in a single test.

#### 2.4.1. Preparation of Wine Samples

Twenty bottles (1 L) of Riesling wine samples with various concentrations of TDN were prepared in the morning before each *sensory session*. The wine was preliminarily homogenized in a stainless steel container and transferred back to the bottles. Wine samples were spiked with the TDN stock solution and mixed in order to reach the target TDN concentrations of 4–202 µg/L according to the design of the experiment (Figure 1). The prepared wine samples were kept in 1 L bottles not more than 2–3 h at room temperature before pouring into the glasses. 

The highest TDN concentration was limited to 202 µg/L. This value was chosen on the basis of the maximal reported *CRejT* being 157 µg/L [23] and included a necessary margin. The TDN content was increased in small steps between 2 and 22 µg/L in the first ten glasses since *DT* and *RT* were expected to be around these values and required a precise determination (Figure 1). For the last ten glasses, the difference of TDN concentration between the wine samples was larger. This was a compromise in order to cover a bigger range of values (22–202 µg/L) for the determination of *RejT* and to keep a reasonable number of glasses in the test.

#### 2.4.2. Performance of Thresholds Determination Test

**Figure 2:**
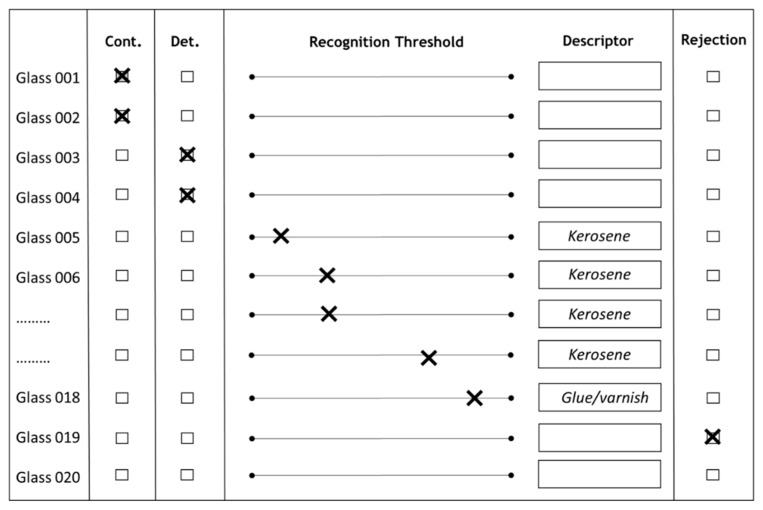
An example of a completed *threshold determination test* questionnaire.

The method of *DT* and *RT* thresholds determination described by Busch-Stockfisch (2002) [28] was reviewed and approved in the Department of Enology, Hochschule Geisenheim University. As a modification to this method, the determination of *RejT* was added and the questionnaire was redesigned (Figure 2). Twenty wine tasting glasses (ISO 3591) containing the corresponding wine samples were placed on the table in front of each panelist. Each glass contained about 35 mL of one of the wine samples poured 30–45 min before the start of the test and immediately covered with a plastic lid. The wine temperature during the sensory evaluation was 22 ± 1 °C. The glasses with serial numbers on the plastic lids were presented in the order of increasing TDN concentration. The panelists were informed that the wine sample in glass #1 was a control sample and each following glass contained the same base wine with the content of TDN equal or higher compared to the previous one ([TDN] “glass n+1” ≥ [TDN] “glass n”). The concepts of *DT*, *RT,* and *RejT* were clarified to the panelists. The task of the test was to evaluate the wine samples orthonasaly one-by-one, starting from the 2nd glass using the paper questionnaire. If the wine in the glass was perceived to be the same as the control (glass #1), an indication should be made in the column “Cont.”. If the following wine sample was different from the control, but no TDN related aromas were recognized, the column “Det.” should be chosen (*detection threshold*). The “Recognition threshold” column was provided for the wine samples in which TDN aroma could be identified (Descriptor) and evaluated by intensity. The last column “Rejection” was introduced for the *rejection threshold*. It was explained as a concentration of TDN, at which aroma intensity was not acceptable (too high and unpleasant) in the bouquet of the wine. The first markings in the “Det.” and “Recognition Threshold” columns were considered as panelist’s personal *DT* and *RT*, respectively. Once judge reached the last column “Rejection”, the test was finished.

During the test, panelists were not allowed to return to the previous glasses. This measure was applied due to the possibility of a panelists’ adaptation to a higher TDN content and changes in the assessment of preceding samples. In addition, it was recommended to do not more than 3–4 sniffs per glass and to agitate the wine sample only after the first sniff. The panelists evaluated the wine samples at an individually convenient pace with pauses between samples, if necessary. No specific training was carried out prior to the *sensory sessions*. However, after the explanation of the test rules, the panelists were asked to do a trial test run, which was followed by a 15 min break. Later, the main test was performed. According to the method [28], the threshold values were determined in two ways: by the lowest value found by 50% of tasters and by the geometric mean value based on the answers of all panelists (Table 2).

### 2.5. 3-AFC Tests

#### 2.5.1. Preparation of Wine Samples

The wine samples preparation for the *3-AFC tests* was based on the *RT* values defined in the *thresholds determination tests*. Each *3-AFC test* comprised two control samples (base wine) and one spiked wine (Figure 1). Four levels of TDN spiking were applied for each *sensory session* in order to reach the following concentrations: “*RT*-5 µg/L”, “*RT*”, “*RT*+5 µg/L”, and “*RT*+10 µg/L”. Two sets of 1 L bottles with the wine samples were prepared in the morning 2–3 h before each *sensory session*. One set of the bottles was kept at room temperature, while the other set was cooled in the refrigerator in order to reach the wine serving temperature of 11 ± 1 °C. 

#### 2.5.2. Performance of 3-AFC Tests

Eight *3-AFC tests* were performed with the same panel within each *sensory session*. Wine samples for the first four tests were served at room temperature (22 ± 1 °C) in order to re-check the *RT* found in the *thresholds determination test.* The last four tests contained the same wine samples as the first ones but at a serving temperature of 11 ± 1 °C. Each glass contained about 35 mL of the wine, which was served immediately before each *3-AFC test*. The panelists were asked the following question: “Which sample has a more intense kerosene/TDN aroma?”.

### 2.6. Processing of the Data 

**Figure 3:**
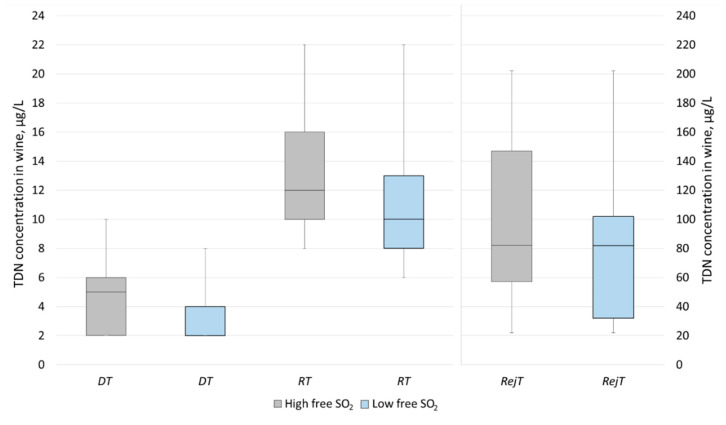
Distribution of the panelists’ answers for the determination of the *detection threshold* (*DT)*, *recognition threshold* (*RT),* and *rejection threshold* (*RejT)* of TDN in Riesling wine. Median is represented by the horizontal line. The bottom and top of the box represent the 1st and the 3rd quartiles, respectively. The whiskers represent the minimum and maximum values.

**Figure 4:**
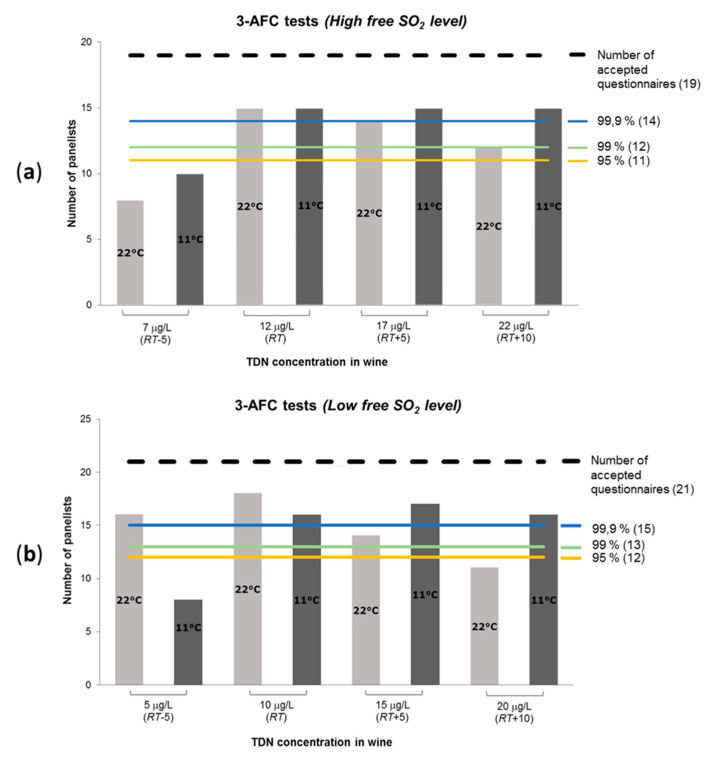
Results of the *3-AFC tests*. The dashed lines represent the total number of panelists whose questionnaires were accepted for the statistical analysis: (**a**) *n* = 19; (**b**) *n* = 21. The colored lines represent the three levels of significance of 95%, 99%, and 99.9% with the number of required panelists (accepted questionnaires).

Only the completely filled out questionnaires were accepted and subsequently statistically analyzed. Therefore, their number in the sensory tests can differ from the total number of panelists (Table 2, Figure 3 and Figure 4). Questionnaires were prepared and processed using Fizz software 2.51a 86 (2016, Biosystemes, Couternon, France). The same software was used for the statistical analysis of data of the *3-AFC tests*. Text, calculations and figures for the *thresholds determination tests* and *3-AFC tests* were done using Microsoft Office Standard 2013 programs (Version 15.0.5153.1000, Microsoft Corporation, Redmond, Washington, DC, USA).

### 2.7. Analysis of TDN Content in Wine

The level of TDN was analyzed in the base wine and validated in the selected spiked wine samples by GC-MS (SBSE) analysis according to the standard operation procedure at the Hochschule Geisenheim University [16]. In particular, the selected samples with TDN content close to the determined *recognition threshold* had the following TDN concentrations (expected/validated): *thresholds determination test* Sample #6 (10 μg/L/9.9 μg/L), Sample #7 (12 μg/L/12.0 μg/L). 

## 3. Results and Discussion

### 3.1. Thresholds Determination Tests (DT, RT, and RejT)

According to the methodology, the TDN sensory thresholds values were determined as the lowest ones reported by 50% of the panelists and by the geometric means. Both methods in the current study revealed close results (Table 2). The box plots are presented for the demonstration of the panelists’ answers distributions (Figure 3). The calculated medians were equal to the 50% panelists values, except for the *DT* (*high* free SO_2_): 5 µg/L and 4 µg/L, respectively.

In general, the sensory thresholds for Riesling wine with *high* and *low* free SO_2_ content were similar. At the same time, a trend towards smaller values was observed in the distribution of panelists’ responses for the wine samples with the *low* free SO_2_ content (Figure 3). 

The determined *detection thresholds* at about 4 µg/L were close to the *ODT* found by the trained panelists at 3.1 µg/L in Riesling wine [24] and somewhat higher than the reported O*DT* in neutral Chardonnay or model wines at 2 µg/L [20]. The latter difference was predictable since Riesling was the base wine in the current study and it initially possessed 2.2 µg/L of TDN. In general, many Riesling wines contain about 4 µg/L of TDN, however, according to the concept of *DT*, it does not mean that these wines have a noticeable kerosene/petrol aroma. In addition, the comparison of TDN sensory thresholds in Chardonnay and Riesling wines is not relevant, since the latter wines are usually much more aromatic. Therefore, the TDN thresholds in Riesling wines are expected to be higher due to the wine matrix effects.

The *recognition thresholds* for the wines with *high* and *low* free SO_2_ content were identified at 12 µg/L and 10–11 µg/L, respectively. These values are almost two times lower compared to the initially reported *flavor threshold* of 20 µg/L [18] or *ODT* at about 18–21 µg/L [23] and slightly lower than the 14.7 µg/L *CDT* defined by consumers in Riesling wines [24]. The minimal and maximal *RT* values indicated by the panelists were similar for both wines with the *high* and *low* free SO_2_ content: 8–22 µg/L and 6–22 µg/L, respectively. At the same time, the distribution of the panelists’ answers within the 1st and 3rd quartiles comprised somewhat smaller values for the *low* free SO_2_ wines compared to the *high* free SO_2_ counterparts: 8–13 µg/L vs. 10–16 µg/L, respectively. An excessive free SO_2_ content in wine may partially mask TDN aroma, especially when the typical smell of sulfur dioxide is perceivable, as it was on the *Sensory Session 1*. 

The *rejection thresholds* of the individual panelists varied significantly between 22 and 202 µg/L in both sessions. Both calculation approaches revealed similar results, about 80 µg/L for the *high* free SO_2_ wine and around 70–80 µg/L for the *low* free SO_2_ samples. These results were comparable with the *CRejT* values found in two 1-year-old Riesling wines at 60 µg/L [24] and 82.4 µg/L, but about two times lower than the other reported *CRejT* value of 157 µg/L [23] (Table 1). The variation of the presented values is not surprising, since the optimal approaches of *rejection threshold* determination remain under discussion. For example, in the recent comment regarding the preference tests for *CRejT* evaluation, it was remarked that even if one sample is not preferred sensorially over another, this does not always mean that a non-preferred sample is rejected [26]. Instead of preference tests, the panelists of the current research were asked to identify when the Riesling wine starts to possess a not acceptable (too high and unpleasant) level of TDN aroma. It is noteworthy that at high TDN concentrations, close to the individual *RejT*, some of the panelists switched from the kerosene/petrol aroma descriptors to those of solvent, glue, varnish, rubber, and pharmacy (Figure 2).

The influence of TDN content on the evaluation of Riesling wines remains to be demonstrated. Among the factors that can affect the acceptance/rejection of elevated TDN content in Riesling wine are different TDN aroma tolerances in various groups of people, regional consumers’ habits, and variability of wine matrices (vintages, young, and aged wines). The vivid demonstration of these effects is the almost twofold difference of *CRejT* values for 1-year-old Riesling wines (157 µg/L and 82.4 µg/L) depending on the vintage (2010 and 2011) and the country in which the sensory tests took place (New Zealand and the USA), respectively, [23]. Another example is the Australian Riesling wine, which despite the great TDN content of 246 μg/L received a high sensory quality score [19]. In addition, it is not excluded that there are other volatile compounds in wine apart from TDN, which can be associated with the kerosene/petrol aroma.

### 3.2. 3-AFC Tests, Confirmation of the Recognition Threshold, and Influence of Wine Serving Temperature on the TDN Aroma Recognition

Since the *recognition threshold* is essential in terms of the wine aroma composition, it was decided to confirm the determined *RT* values with an alternative sensory method. The utilized *3-AFC tests* implied comparison of the spiked wines with the control samples according to the intensity of the kerosene/TDN aroma perception. The wine samples spiking was designed to reach the TDN concentrations below, above, and equal to the defined *RT* (Figure 1 and Figure 4). The serving temperature of the wine was the same as for the *thresholds determination tests*, i.e., 22 ± 1 °C. Additionally, the identical *3-AFC tests* were conducted at a lower serving temperature, 11 ± 1 °C, in order to check whether the TDN recognition is temperature-dependent. 

The results of the *3-AFC tests* demonstrated that all the spiked samples at the level of *RT* (12 µg/L with *high* and 10 µg/L with *low* free SO_2_ content) were statistically significantly different from the control samples (Figure 4). At the same time, the panelists were not able to distinguish the “*RT*-5” spiked samples from the control wines at *high* free SO_2_ level, which confirms that the *RT* was higher than 7 µg/L. In the case of *low* free SO_2_ wine, the tasters identified the spiked wine “*RT*-5” at room temperature (99.9% significance). However, it does not mean that the *RT* for the wine with *low* free SO_2_ was 5 µg/L. The panelists were able to identify the spiked sample, but not necessarily recognized that it had the kerosene/petrol aroma, i.e., the effect of *detection threshold*. This suggestion is supported by the results of the *3-AFC test* at low temperature, whereby the “*RT*-5” spiked wine was not distinguished from the control samples at both levels of free SO_2_ contents.

The panelists’ ability to distinguish TDN aroma at concentrations ≥ *RT* at lower temperature was always highly significant, 99.9%. At the same time, the recognition of the spiked wine samples at room temperature dropped down in the sequence of tests with the TDN concentrations “*RT*”, “*RT*+5”, and “*RT*+10”. This phenomenon can be related to olfactory fatigue (sensory adaptation), but not exclusively, since the same effect was not observed at the lower wine serving temperature. The other possible reason is the particularity of wine aromas’ volatility at different temperatures. Thus, the air in the glass at a lower temperature should be enriched with hydrophobic molecules such as TDN, which are more volatile compared to hydrophilic compounds of similar molecular weight. Hence, this should facilitate the sensorial recognition of the TDN aroma. In the case of a higher temperature, the volatility of all wine aroma components rises, but not proportionally. Therefore, the fraction of TDN with regard to other volatile compounds in the air inside the glass can decrease, which complicates the sensorial identification of the TDN aroma. The results of this phenomena were also observed during the scalping process of TDN by wine stoppers, whereby TDN was absorbed noticeably faster at lower temperatures in the vertical bottle position [16]. In addition, some aroma compounds that become sensorially noticeable only at higher temperatures can also cause certain masking effects. 

## 4. Conclusions

The modified *thresholds determination method* demonstrated a convenient approach to define three sensory thresholds of TDN in the Riesling wine in one run: *detection threshold* (*DT*), about 4 µg/L, *recognition threshold* (*RT*), 10–12 µg/L, and *rejection threshold* (*RejT*), 71–82 µg/L. The *RT* values were additionally confirmed by the series of *3-AFC tests*. In comparison with the earlier defined TDN sensory thresholds, the current *RT* was somewhat lower than the previously reported *consumer detection threshold*, while the determined *RejT* values were close to some of the recently published *CRejT* values (Table 1). Nevertheless, no direct comparison of the latter values can be done since the wine acceptance/rejection concepts were diverse in different studies. 

Variation of free SO_2_ content in the wine did not affect substantially the TDN sensory thresholds, however, the noticeable smell of sulfur dioxide at *high* level of free SO_2_ tended to mask the perception of the kerosene/petrol aroma. Finally, it was shown that the TDN aroma recognition was easier in cooled wine, about 11 °C, which is likely related to the particularities of odorants’ volatility depending on temperature.

## Figures and Tables

**Table 1:** Summary of the studies devoted to the 1,1,6-trimethyl-1,2-dihydronaphthalene (TDN) sensory thresholds determination.

References	Panel	Base Wine	Wine Temperature	Sensory Method	Sensory Thresholds, µg/L
[18]	n/a	Riesling	n/a	triangle tests	*Flavor threshold:* 20
[20]	Trained	Model wine; Chardonnay	n/a	3-AFC tests	*ODT*: 2
[23]	Untrained consumers	Riesling, 1-year-old wine	23 °C	3-AFC tests	*ODT*: 20.6 (2010 vintage, NZ), 18.2 (2011 vintage, USA) *CRejT*^1^: 157 (2010 vintage, NZ), 82.4 (2011 vintage, USA)
[24]	Trained consumers	Riesling, 1-year-old wine (2015 vintage)	15 °C	3-AFC tests	*ODT*, Trained panel: 3.1 *CDT*, Consumers: 14.7 *CRejT*^1^ 1-year-old wine: 60 *CRejT* ^1^ 8-year-old wine: 91

^1^*CRejT* was determined by the preference tests.

**Table 2:** Results of the thresholds determination tests.

Sensory Sessions (Accepted Questionnaires)	Thresholds	Calculation Approaches		
		Geometric Mean	50% Panelists	Median
Session 1	*Detection* (*DT*)	4 µg/L	4 µg/L	5 µg/L
high free SO_2_,	*Recognition* (*RT*)	12 µg/L	12 µg/L	12 µg/L
(*n* = 16)	*Rejection* (*RejT*)	79 µg/L	82 µg/L	82 µg/L
Session 2	*Detection* (*DT*)	3 µg/L	4 µg/L	4 µg/L
low free SO_2_,	*Recognition* (*RT*)	11 µg/L	10 µg/L	10 µg/L
(*n* = 20)	*Rejection* (*RejT*)	71 µg/L	82 µg/L	82 µg/L
